# Investigating the psychological impact of COVID-19 on Rob Ferreira Hospital healthcare workers in Mpumalanga Province, South Africa

**DOI:** 10.3389/fpsyg.2025.1553866

**Published:** 2025-07-29

**Authors:** Tlou D. Raphela, Magagula Happiness

**Affiliations:** Disaster Management Training and Education Centre for Africa, University of the Free State, Bloemfontein, South Africa

**Keywords:** psychological impact, healthcare workers, COVID-19 pandemic, mental health, burnout, resilience

## Abstract

**Introduction and aim:**

The psychological impacts of the COVID-19 pandemic on healthcare workers (HCWs) were profound and far-reaching. This study, therefore, investigated these effects among HCWs at Rob Ferreira Hospital in Mpumalanga Province, South Africa, focusing on mental health measures that include stress, anxiety, depression, burnout and compassionate fatigue experienced amid the COVID-19 pandemic with the aim to determine existing coping mechanisms and institutional support systems.

**Methodology:**

Using a quantitative research approach, data were collected through semi-structured questionnaires and analyzed quantitatively for 100 HCWs who participated, providing a comprehensive perspective on their psychological well-being during the pandemic.

**Results:**

Key findings revealed shows the intersectionality of mental health vulnerability, showing how language and caregiving roles interact with the emotional burden of a disaster. Therefore, targeted mental health interventions should focus on providing linguistically accessible services and outreach programs. The results also highlight the critical role of staff support in influencing how individuals cope and how effective they perceive their coping methods. The study found that institutional support systems were perceived as insufficient, with counseling services not being adequately utilised, contributing to increased frustration and resentment toward management. Additionally, coping mechanisms provided by the hospital, including peer support groups, were deemed ineffective by most participants.

**Discussion and conclusions:**

The study concluded that the psychological well-being of HCWs required immediate and sustained attention. The implementation of structured mental health programs, including regular debriefing sessions, easily accessible counseling services, and stress management training, is recommended by this study. This study conclude that there is weak evidence of institutional support affecting HCWs’ training and preparedness to use mental health systems during COVID-19.

## 1 Introduction

The Severe Acute Respiratory Syndrome Coronavirus 2 (SARS-CoV-2) virus eruption and its related illness, termed COVID-19, led to an unparalleled worldwide health crisis (Ransing et al., 2020). Considered by the World Health Organization (WHO) as a global pandemic on 11 March 2020, the COVID-19 pandemic led to a series of after-effects, with countries following each other with lockdown measures to introduce control measures to curb the spread of the pandemic. Ransing et al. (2020) reported that the pandemic spread led to countries reporting higher mortality rates while dealing with enormous economic losses, social disturbances, and social distancing.

The pandemic also increased concerns about the possibility of a prevalent surge in mental health challenges (Bhatia et al., 2021; Fegert et al., 2020; Hossain et al., 2020). Shaukat et al. (2020) state that healthcare workers (HCWs) have been psychologically impacted by working daily for long hours since the start of COVID-19, and their exposure to this virus has been very high. Furthermore, HCWs encountered high levels of depression, insomnia, anxiety, and distress, which COVID-19 can exacerbate. Sethi et al. (2020) highlight that during COVID-19, health professionals were overworked, financially unstable, and anxious while creating, planning, and providing care to others, including their families. Furthermore, the rapid spread of the pandemic was often underestimated, prompting nations to adopt a reactive stance in addressing the crisis.

The virus had profound repercussions on healthcare service delivery to patients severely impacted by the pandemic, while HCWs faced significant challenges in multiple dimensions (Pak et al., 2020). Owais and Shehzad (2024) elucidate that pandemics, by their nature, pose unprecedented mental health challenges for HCWs. They elaborate that HCWs, as frontline responders, frequently experience anxiety, burnout, depression, insomnia, and other stress-related disorders. Additionally, absenteeism, depression, and other psychological issues may arise from the loss of colleagues, family members, and patients, contributing to increased rates of mortality among HCWs due to compassion fatigue and vicarious traumatization. Norhayati et al. (2021) supports this perspective, highlighting that in the battle against the COVID-19 pandemic, HCWs have encountered intense pressure stemming from heightened infection risks, inadequate protective measures against contamination, and insufficient support in managing the stressors associated with workload, frustration, and exhaustion.

Smallwood et al. (2022) indicate that the epidemiology of the COVID-19 pandemic among healthcare workers (HCWs) is continuously being explored, with many identified risks leading to COVID-19 being regarded as an occupational illness for HCWs. Furthermore, Smallwood et al. (2022) explain that similar to the general population, HCWs have experienced long-term illnesses due to COVID-19, which have negatively impacted their return to work. The HCWs have also been significantly affected by the substantial workplace and psychological disturbances arising from the pandemic. Moreover, the impacts on the psychological well-being of HCWs are profound, with high incidence estimates of mental health symptoms, including emotional exhaustion. Barello and Graffigna (2020) indicates that there are various intervention strategies aimed at alleviating emotional distress among HCWs who are vulnerable to contracting the virus through outbreaks.

The observation made by Barello and Graffigna (2020) underscores the urgent need to develop strategies to protect and promote the mental well-being of HCWs during and after the outbreak. Blake et al. (2020) have focused on mitigating the psychological impact of COVID-19 on healthcare workers through a digital intervention package. Conversely, investigating the psychological effects of COVID-19 is crucial for the implementation of disaster risk reduction, as outlined by the Hyogo Framework for Action (2005) (HFA) and the current Sendai Framework for Disaster Risk Reduction (2015) (SFDRR). Priority number two of the HFA emphasizes the necessity of identifying, assessing, and monitoring disaster risks and improving early warning systems (Hyogo Framework for Action, 2005). Understanding the psychological impact of COVID-19 on healthcare workers can help avert numerous challenges that may arise within the health system, subsequently affecting communities. Different countries adhere to international frameworks on disaster risk reduction, such as the Sendai Framework, which assigns the responsibility of disaster risk reduction to national and local governments while obligating global and regional levels to fulfill their roles. The HFA primarily focuses on risk identification and governance, which will be examined in this study, although other priorities outlined in the current SFDRR will also be addressed.

This study, therefore, aims to investigate specifically the psychological impact of COVID-19 on healthcare workers at Rob Ferreira Hospital by answering the following questions: (1) What are the mental health symptoms caused or exacerbated by the COVID-19 pandemic amongst the HCWs; (2) How effective were existing support systems and coping mechanisms utilised by healthcare workers at Rob Ferreira Hospital in mitigating the psychological effects of the COVID-19 pandemic. The study sub-objectives focuses on stress, anxiety, depression, and burnout and compassionate fatigue experienced during the COVID-19 pandemic. Evaluated existing coping mechanisms and support systems within the study hospital.

## 2 Study methodology

### 2.1 Study area description

Rob Ferreira Provincial Hospital is a public healthcare facility administered by the Department of Health of the Mpumalanga Province. The hospital is located in Mbombela local council, under Ehlanzeni District Municipality in Nelspruit, Mpumalanga. As a provincial hospital, Rob Ferreira Hospital provides complimentary services to breastfeeding and expectant women and children under the age of six. Other residents are charged fees following government-prescribed hospital rates. The hospital also offers a range of specialist services, including but not limited to eye care, family medicine, primary healthcare, obstetrics, psychiatry, internal medicine, geriatrics, surgery, and rehabilitation. The study adopted a quantitative approach. This approach incorporates descriptive and inferential statistics.

### 2.2 Target population and sampling

Willie (2022) reported that a target population is a comprehensive subsection of the population of interest in research. Rob Ferreira Hospital, the targeted hospital, has 300 clinical staff members. This study’s sample size of 169 was calculated from this targeted population of 300 using a sample size calculator with a confidence interval of 95, 5% margin of error and 50% population proportion. However, data used for this study was from 100 HCWs, as some of the 69 questionnaires were not usable (49), most critical questions especially on mental health and Likert scale were not answered. 20 HCWs did not return their questionnaire. Indeed, healthcare workers were reported to be COVID-19 fatigued in 2023 (Hui et al., 2023) and we therefore decided to use the 100 questionnaires to answer the objectives of this study. Hopkins (2017) reported that a sample size of 100 is enough to make statistical inferences.

The sample size inclusion criteria were (a) the practicality of speaking to healthcare workers while they were on duty, (b) inadequate resources (human and financial), and (c) time constraints to survey all 300 healthcare workers because most HCWs work shifts. A convenience sampling technique was utilised for this study.

### 2.3 Data collection and analysis

The data for this study was collected using a semi-structured questionnaire developed by the authors. The questionnaire was approved and ethically cleared by the General Human Research Ethics Committee of the University of Free State under protocol number (UFS-HSD2023/1503). The questionnaire comprised of close-ended questions divided into four sections, namely demographics, mental health measures, Institutional support and coping mechanisms focusing on the assessment of mental health symptoms among HCWs of Rob Ferreira Hospital amid the COVID-19 pandemic.

The data was captured and uniquely coded for different variables for each questionnaire using Microsoft Excel. In addition, the study analysed the data employing inferential statistics using R Statistical Package Software statistics. Data for this study was not normally distributed therefore we employed nonparametric statistical tests to analyse the data. Statistical tests were two-tailed, and we set the *p*-value to ≤0.05 with 95% confidence interval. Data for this study was visualized using Figures produced using GGplot2 from the R software and tables were produced using Microsoft Excel. Furthermore, the statistics assisted in illuminating the correlation between measurable variables.

#### 2.3.1 Statistical analysis

To address the objectives of this study, we first analysed some the demographics of the Healthcare workers descriptively to put the study into perspective in terms of the profile of the HCWs who could be at risk of pandemic based on age, gender, title, years being employed at the hospital and the department that the HCWs are primarily responsible for. Secondly, the study applied five separate Chi-squared tests of independence to establish the relationship between the some of the critical demographic questions and the mental health measures.

This study is undertaken within the Disaster Management context, and in Disaster Management training is one of the important variable in the disaster preparedness framework. We therefore applied a logistics regression to Thirdly to address the institutional support objective of this study we applied a logistics Regression to assess the support that the HCWs have received from their employers and set the statement “I have been adequately trained and equipped to utilize the available support systems for addressing my mental health concerns amid the COVID-19 pandemic.” as a response variable and regressed this statement against four other statements (set as independent variables for the regression model) that will answer the support that the HCWs received amid the pandemic. The responses for these statements were set as “Yes” or “No” and coded as “1” and “2” to make the regression possible. Lastly this study assessed the coping strategies employed by the HCWs of the study hospital by running a series of Spearman’s Rank correlation simultaneously for the four Likert scale statements from the study Questionnaire (Supplementary Material 1) to assess the correlations between variables.

### 2.4 Data validity and reliability

The reliability statistics in Table 1, assessed using McDonald’s *ω* (omega) and Cronbach’s *α* (alpha), provide insights into the internal consistency of the scale determined using SPSS. The point estimate for McDonald’s ω is 0.778, suggesting a strong level of internal consistency, as values above 0.7 are generally considered acceptable for reliability. The 95% confidence interval (CI) for *ω*, ranging from 0.766 to 0.790, is narrow, indicating stability in the reliability estimate and reinforcing that the scale consistently measures the intended construct. Similarly, Cronbach’s *α* has a point estimate of 0.754, indicating acceptable internal consistency, although slightly lower than McDonald’s *ω*. This value remains above the 0.7 threshold, suggesting that the items within the scale are sufficiently correlated to assess a unidimensional construct. The 95% CI for *α*, between 0.741 and 0.767, is also narrow, confirming the consistency of this reliability measure. McDonald’s *ω* and Cronbach’s *α* suggest that the scale has adequate internal reliability, with point estimates above 0.75 and consistent confidence intervals for both metrics. These values indicate that the scale is likely a reliable tool for measuring the intended construct within the sample.

**Table 1:** Data reliability statistics.

Estimate	McDonald’s *ω*	Cronbach’s *α*
Point estimate	0.778	0.754
95% CI lower bound	0.766	0.741
95% CI upper bound	0.790	0.767

## 3 Results and discussions

### 3.1 Characteristics of the respondents

The age distribution data for the respondents with 100 valid responses gave the mean age of 37.56 years, indicating that the average age of individuals in this group is in their late 30s. The standard deviation of 7.488 shows a moderate spread around the mean, suggesting age variability, though not excessively wide. The minimum age recorded is 28 years, and the maximum is 51 years, giving a range of 23 years across the sample. This range indicates a mixture of younger and older adults within the workforce. However, the average and standard deviation imply that most individuals fall within a few years of the mean age. This age profile suggests a mature workforce, predominantly in the early to mid-career stages, with a distribution centered around the late 30s. These mid-career HCWs of up to 51 years of age were reported by a systematic review study as being of less risk to the pandemic as compared to the older HCWs of 60^+^ (Starke et al., 2021).

The results in Table 2 reveal notable gender distributions across healthcare job titles, years of experience, and Departments where respondents were employed, suggesting a disparity favoring female representation in this role (Table 2). The trend is even more pronounced for pediatric doctors, where all 15 positions are held by females, leaving no male representation in this specialty. This could indicate a strong preference or trend for females in pediatric specialties within this context. The role of registered professional nurses shows a similarly skewed gender distribution, with 59 out of 70 (84.3%) nurses, the majority of the sample, being female (Table 2). This pattern aligns with broader healthcare trends, where nursing is often more female-dominated (Wang et al., 2020). With a *p*-value above the standard significance level of 0.05, this result suggests that the observed gender differences across job titles are not statistically significant and could be due to chance rather than a systematic difference in gender distribution across these roles.

**Table 2:** Gender distributions across healthcare job titles, years of experience, and departments (ICU, intensive care unit).

Gender levels	Variables	Number of responses	*χ*^2^ results
Job title			
Female	Medical doctor	11	*χ*^2^ = 4.276; *p* = 0.118
Female	Pediatric doctor	15	
Female	Registered professional nurse	59	
Male	Medical doctor	4	
Male	Pediatric doctor	0	
Male	Registered professional nurse	11	
Years of experience			
Female	10 years	13	*χ*^2^ = 54.622; *p* < 0.001
Female	12 years	24	
Female	15 years	15	
Female	17 years	15	
Female	3 years	3	
Female	5 years	15	
Male	10 years	0	
Male	12 years	4	
Male	15 years	0	
Male	17 years	0	
Male	3 years	11	
Male	5 years	0	
Departments			
Female	Casualty	24	*χ*^2^ = 8.402; *p* = 0.038
Female	ICU main	13	
Female	Neurosurgeon	33	
Female	Paediatric ward	15	
Male	Casualty	4	
Male	ICU main	0	
Male	Neurosurgeon	11	
Male	Paediatric ward	0	

### 3.2 Mental health measures and demographics

**Figure 1:**
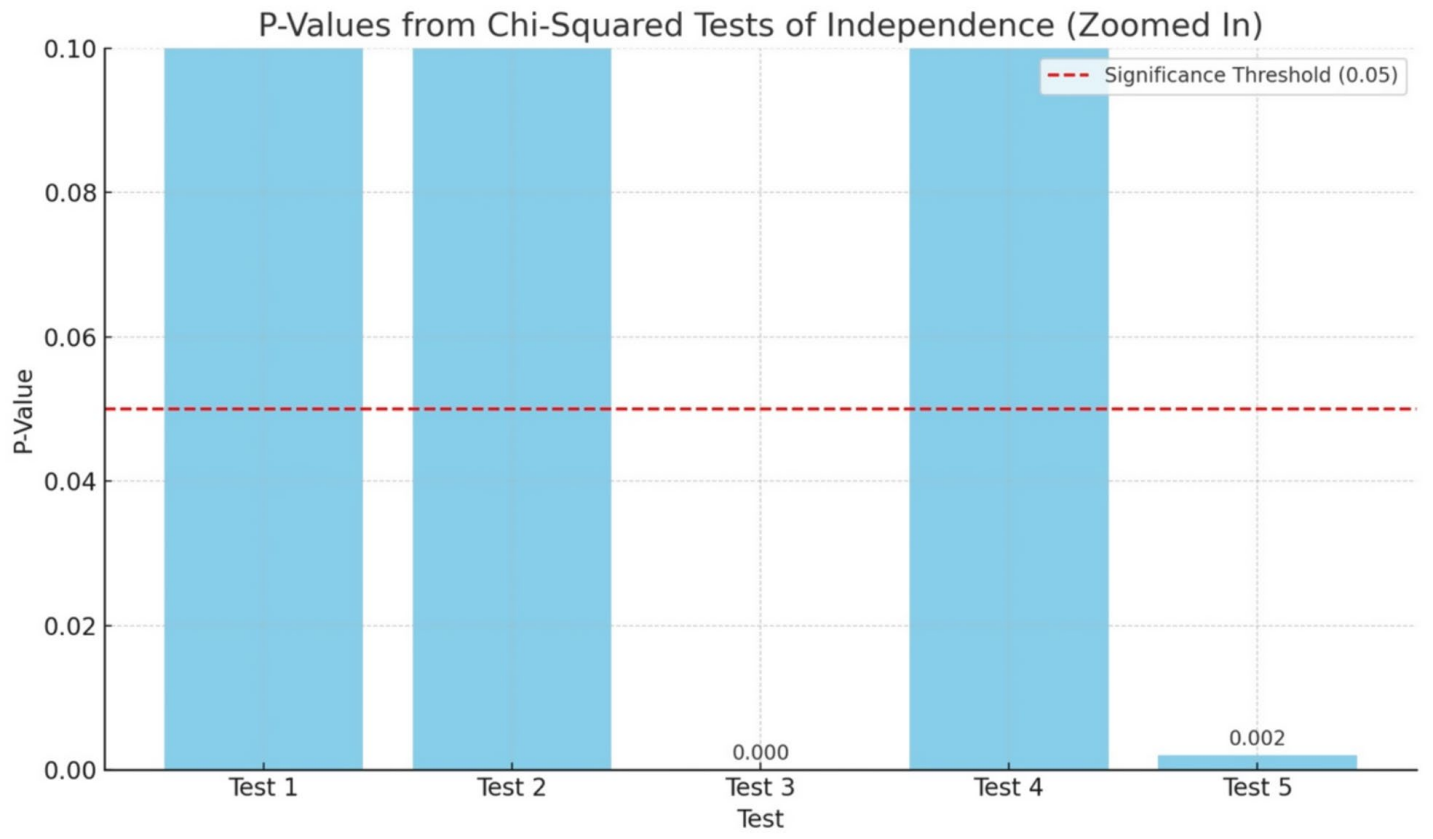
Showing the significant values and significant threshold (red dotted line) for the five Chi Squared tests of independence applied to assess mental health measures and some demographic variables of the HCWs.

The chi squared test of independence showed a statistically significant relationship for anxiety and language spoken by the HCWs (*p* = 0.000) as well as for compassionate fatigue and the dependents that the staff reported to have at home which were children and parents (*p* = 0.002). However, no statistically significant differences were found for Stress and Education; Depression and Marital Status, and Burnout and Employment Status (Table 3, Figure 1). Statistically insignificant figures does not necessarily mean that these factors are not important in predicting mental health, but suggest that in this sample, the factors may not independently predict mental health symptoms in a statistically detectable way.

**Table 3:** Correlation between mental health measures and demographics during the COVID-19 pandemic.

Variables correlated	*χ*^2^ statistic	DF	*p*-value
Stress*education	0.093	2	0.954
Depression*marital status	3.809	4	0.432
Anxiety*language	**4.743**	**3**	**0.001**
Burnout*employment type	4.097	2	0.128
Compassionate fatigue*dependent	**1.366**	**1**	**0.002**

Significant values are shown in bold.

**Figure 2:**
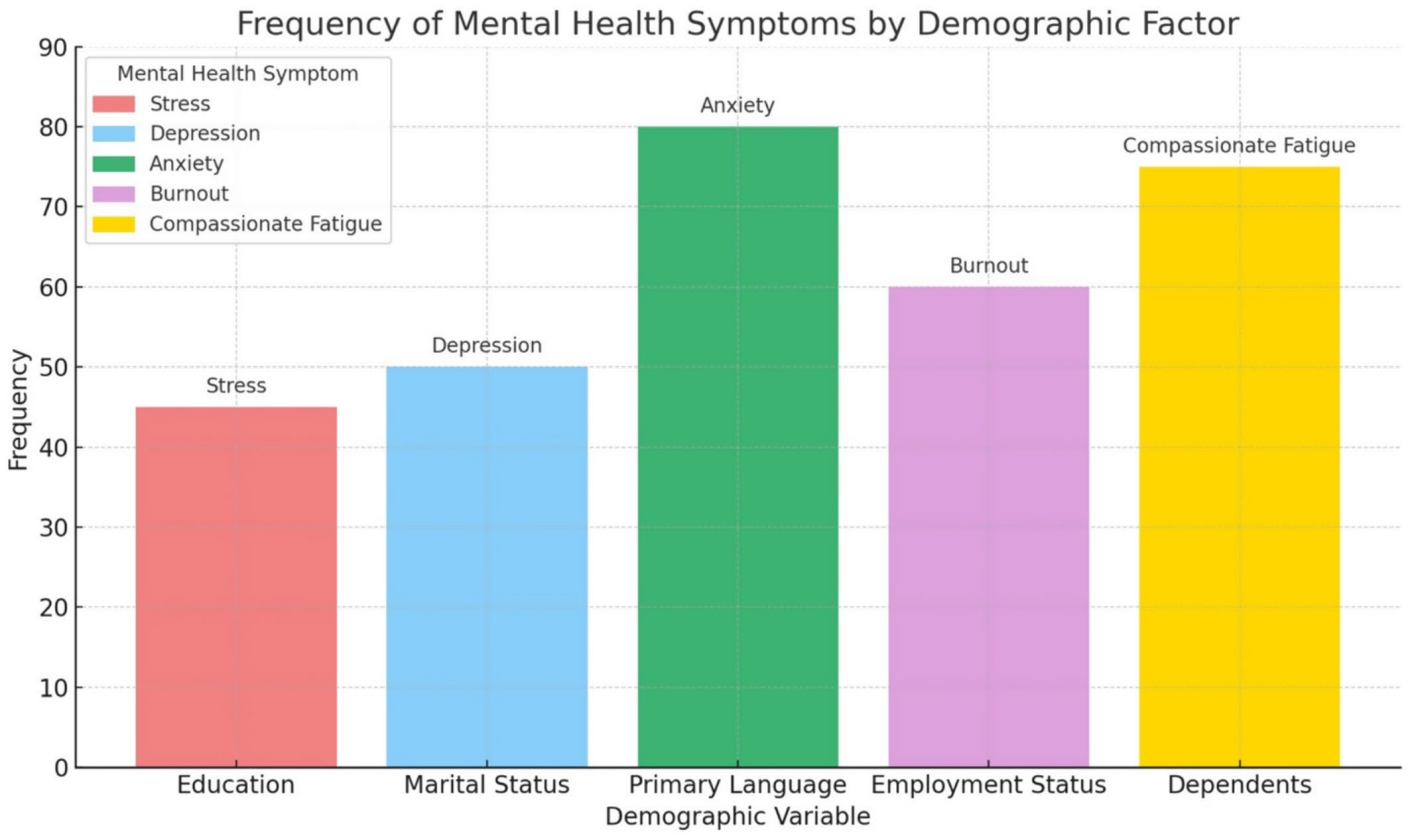
Showing the frequency of mental health measures by demographic variables of healthcare workers.

The frequency distribution of mental health symptoms reported during the COVID-19 pandemic across five demographic categories by the HCWs shows the most frequently reported symptom as anxiety, with 80 participants identifying it, associated with their primary language. This was followed by compassionate fatigue (*n* = 75) linked to the presence of dependents, and burnout (*n* = 60) associated with employment status. Depression and stress had the lowest frequencies, at (*n* = 50) and (*n* = 45), associated with marital status and education level, respectively (Figure 2).

**Figure 3:**
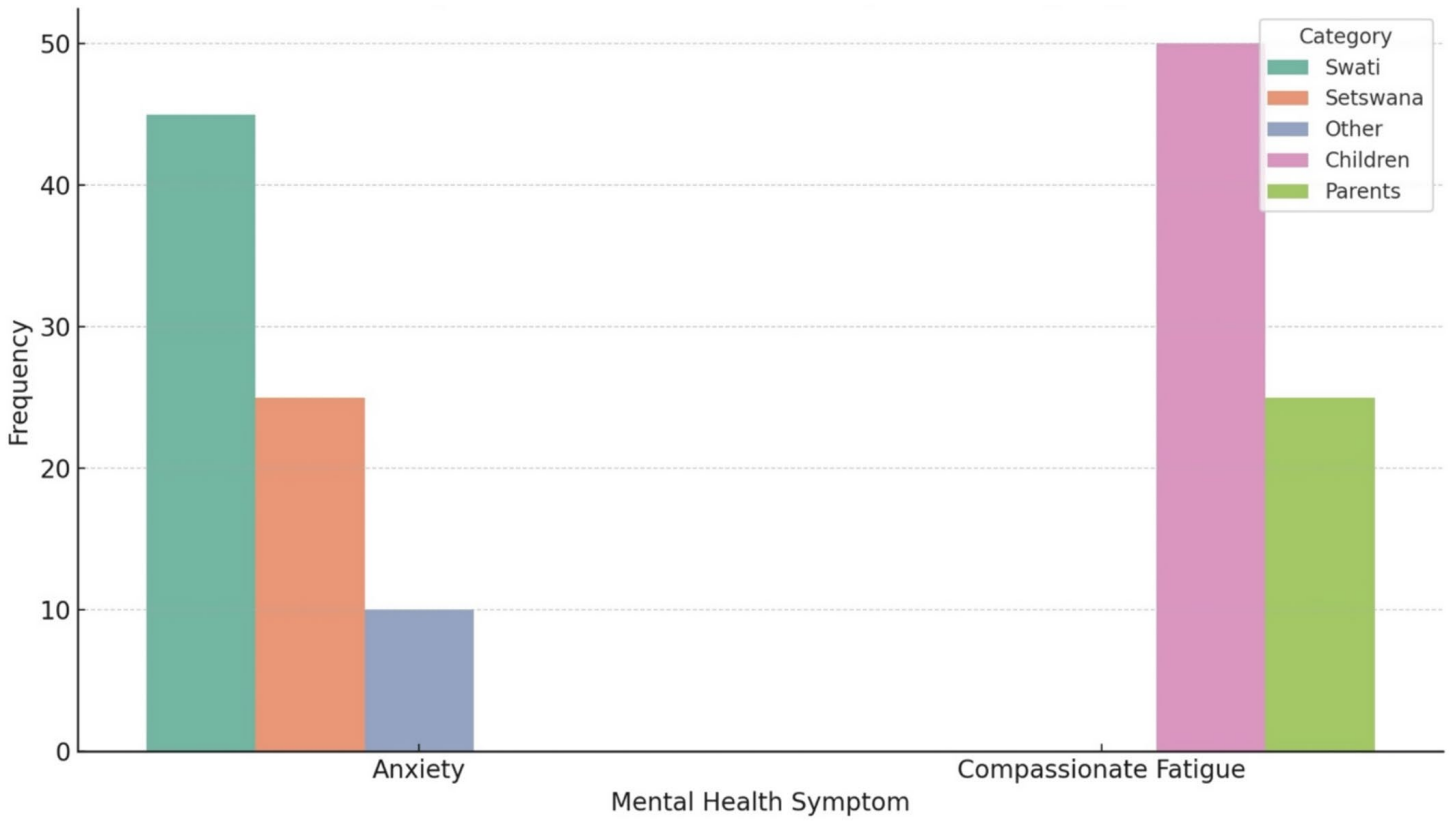
Anxiety and compassionate fatigue by demographic groups: language and dependent.

Figure 3 below presents a disaggregated view of two mental health symptoms, Anxiety and Compassionate Fatigue based on specific demographic variables found to be statistically significant in from five Chi-squared tests of independence that were applied for this study as follows.

#### 3.2.1 Anxiety by primary language

The frequency of reported anxiety symptoms was highest amongst respondents who primarily speak Swati (*n* = 45) followed by Setswana (*n* = 25), and other languages (*n* = 10; Figure 3). This trend suggests that Swati-speaking individuals may be disproportionately affected by anxiety, potentially due to limited access to culturally or linguistically appropriate mental health resources during the COVID-19 pandemic. This could also imply that the Swati HCWs were more anxious as they might have a heighten anxiety level that they might understand the local language better that the other HCWs as the patients especially from the rural areas might express their fears and pains more and fluently with the Swati HCWs as compared to when speaking to other HCWs who are not proficient in Swati. Indeed the study hospital is predominantly in a Swati speaking area and COVID-19 affected everyone even the illiterate. Lalla-Edward et al. (2022) reported that breakdown in communication between HCWs and COVID-19 patients in the rural Limpopo Province. The linguistic barrier was reported to hinder effective communication, coping, or help-seeking behavior, thereby intensifying emotional distress (Lalla-Edward et al., 2022).

#### 3.2.2 Compassionate fatigue by type of dependents

Respondents who had children as dependents reported a significantly higher frequency of compassionate fatigue (*n* = 50) compared to those caring for parents (*n* = 25). A quarter of the respondents (*n* = 25) did not respond to this question (Figure 3). This is a quarter too many and this study will recommend future study to investigate the none responsiveness of these HCWs to the question of compassionate fatigue. However, this finding could underscore the emotional toll on individuals juggling caregiving responsibilities for young dependents during a prolonged public health crisis. The intensity of parental caregiving during lockdowns especially with school closures, remote learning, and health concerns may have elevated the risk of emotional exhaustion that the HCWs were not prepared to share during the time of the study. In Canada Ontario, Dearborn (2024) reported that the pandemic exacerbated compassion fatigues level amongst HCWs. The study also highlighted the unique struggles that HCWs potentially faced as caregivers and how they were able to balance the ever changing pandemic demands with their work and personal life as caregivers and home and frontliners at work (Dearborn, 2024).

### 3.3 Institutional support

We regressed the statement “I have been adequately trained and equipped to utilize the available support systems for addressing my mental health concerns amid the COVID-19 pandemic.” With statements that addresses institutional support of HCWs by their employers (See Table 4; Supplementary Material 1). However, the logistics regression analysis did no show a statistically significant results (*R*^2^_=_0.038; *p* = 0.448). Only 3.8% of the variance in the response variable is explained by the model. Only promotion of a culture of self-care and mental well-being shows a marginal effect of 0.08 (Table 4) suggesting some possible association between the promotion of a culture of self-care and mental well-being and HCWs feeling adequately trained or equipped, though this does not meet the conventional 0.05 threshold for significance that this study has also set. This study results therefore, does not show a sufficient statistical evidence to conclude that institutional support had a strong impact on HCWs perceived preparedness to address mental health concerns.

**Table 4:** Output of a multiple logistics regression for HCWs Institutional support amid the COVID-19 pandemic.

Predictor	Coefficient	*p*-value
Assistance and support	−0.0522	0.670
Effectiveness of existing support system	−0.0569	0.669
Encouragement of open communication and feedback	−0.0334	0.792
Promotion of a culture of self-care and mental well-being	0.2558	0.080

**Figure 4:**
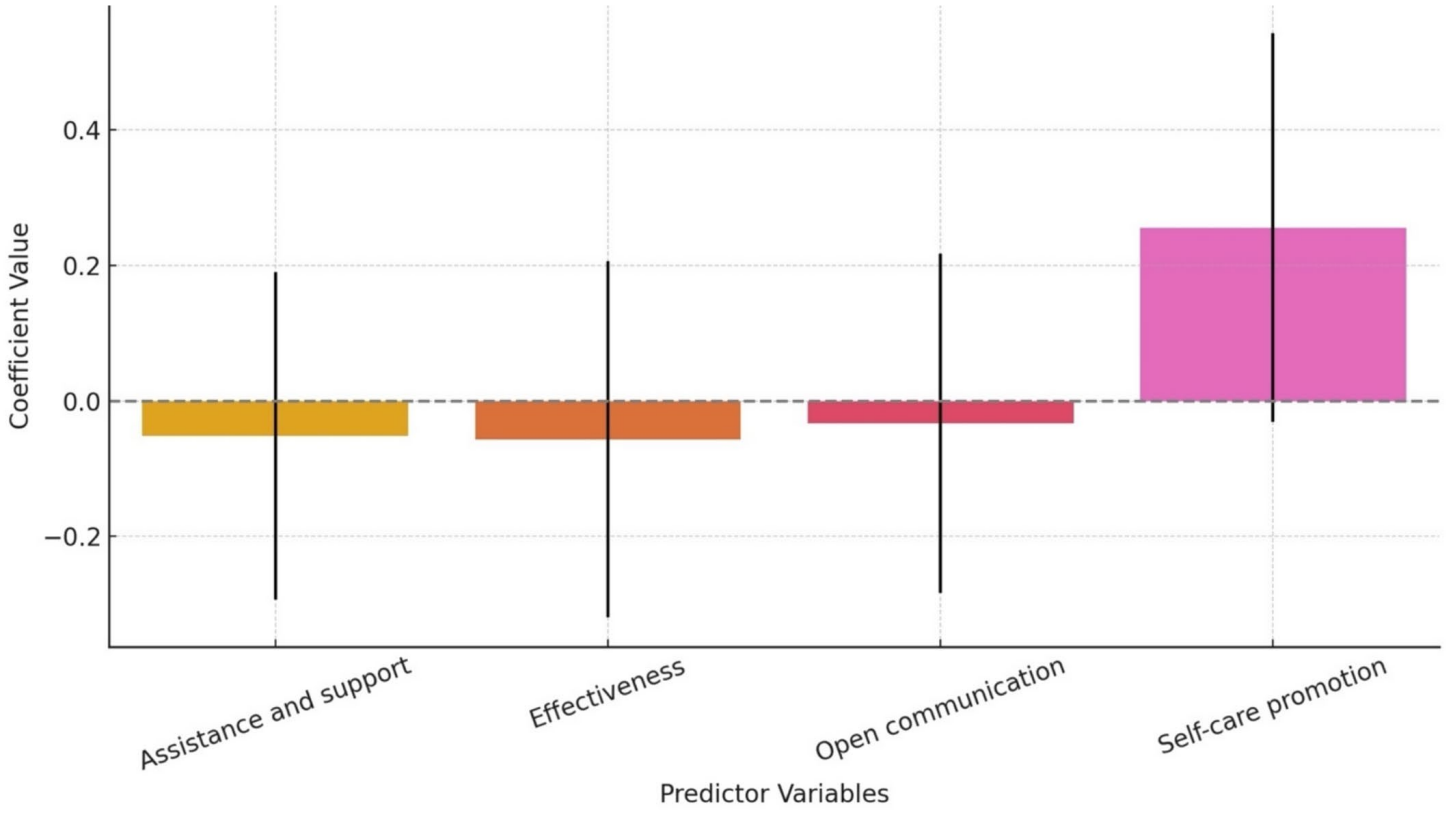
Estimated effects of four predictor variables on HCWs’ perceived preparedness and training amid the COVID-19 pandemic.

Figure 4 shows self-care promotion exhibiting the strongest positive coefficient (~0.26) across all predictor variables. This finding suggests that when the study institution promote self-care and mental well-being, the HCWs are more likely to report feeling trained and equipped to handle mental health challenges amid the COVID-19 pandemic. However, the confidence interval shown by the error bars show that this finding was only marginally significant therefore the results should be interpreted cautiously.

Our study results shows a disconnect between institutional support mechanisms and the perceived readiness of HCWs to use mental health resources amid the pandemic. Although the study hospital may have had support systems in place, most element such as direct assistance, system effectiveness and open communication about mental health did not significantly influenced whether HCWs felt adequately prepared or trained. The area that showed a potential impact was promotion of self-care and mental well-being hinting that cultural or environmental aspects of support may matter more than formal systems. This results means that HCWs in our study may respond positively to visible, encouraging efforts that promotes well-being as part of daily practice rather than reactive or system-based support. Our findings are consistent with a lot of literature suggestion that support and organisational culture often overweigh the mere existence of resources in determining mental health outcomes and resilience among frontline workers (Gupta and Sahoo, 2020; Faculo, 2022; Pollock et al., 1996). Indeed Pollock et al. (1996) reported that HCWs could be supported during disease epidemics by workplace interventions to support basic daily needs this talks to daily practices support. In Guam memorial Hospital in the United State, Faculo (2022) reported that strategies and initiatives that HCWs may implement in the shared responsibility of designing support programs based on meeting the unique needs of HCWs during and after the COVID-19 pandemic are necessary. Furthermore, Gupta and Sahoo (2020) reported that in India, tangible support from the administration was needed by the HCWs amidst the pandemic.

### 3.4 Healthcare workers coping mechanism

The spearman Rank association test shows that staff support appears to be meaningfully associated with both coping strategies and the HCWs perceived benefit. However, counselling shows almost no correlation with the other variables, suggesting it may influence or relate to different factors not captured in these variables (Table 5).

**Table 5:** Spearman correlation output for the coping strategies of the healthcare workers.

Variables	Correlation (*ρ*)	Interpretation
Staff support vs. coping strategies	0.43	Moderate positive correlation
Staff support vs. strategies beneficial	0.45	Moderate positive correlation
Coping strategies vs. strategies beneficial	0.29	Weak to moderate positive correlation
Counselling code vs. others	0.00–0.12	Virtually no correlation

**Figure 5:**
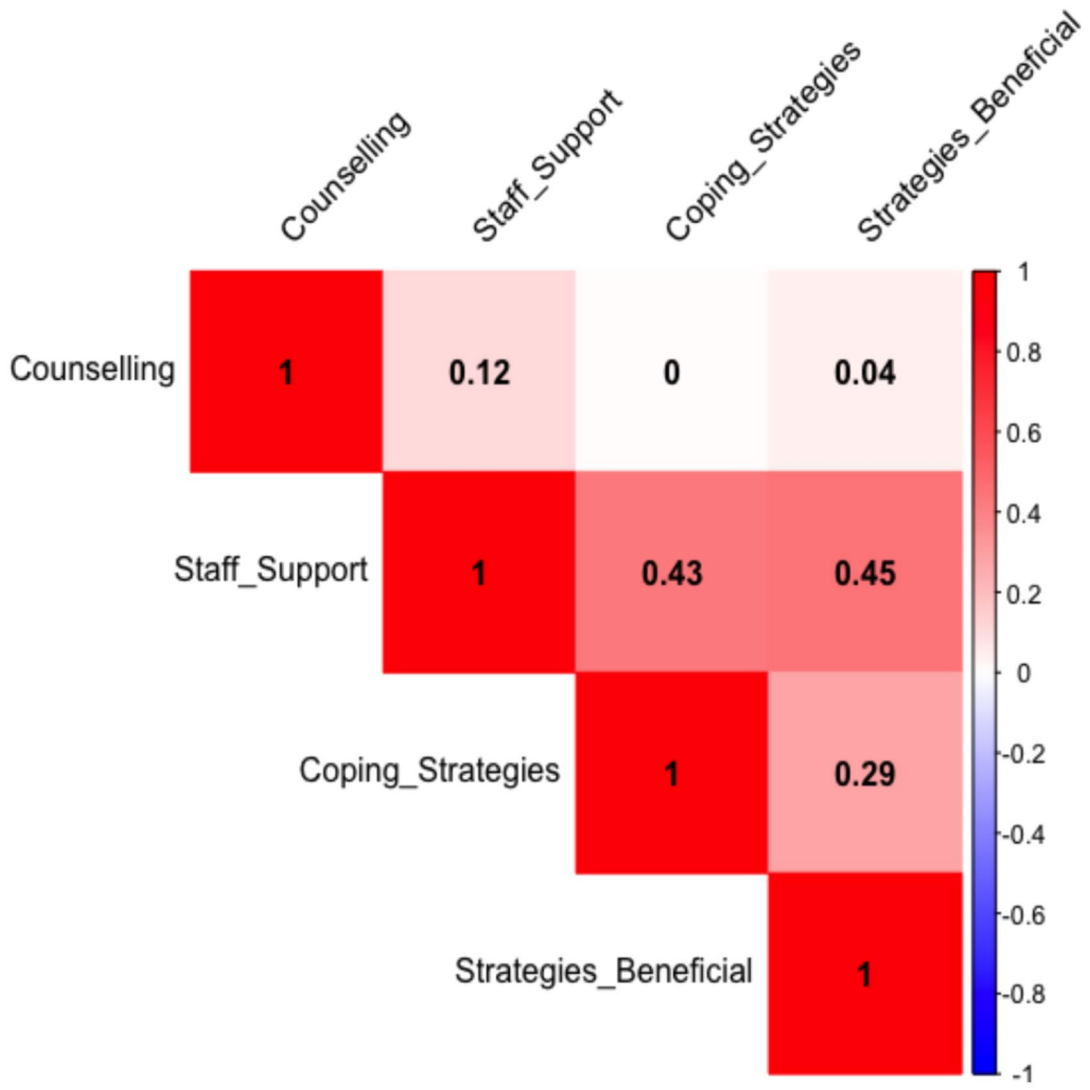
Spearman Correlation heatmap to show distribution and associations of variables for coping strategies of the Healthcare workers.

The heatmap shows the Spearman correlation between four numeric variables (Likert scale codes) for the four statements that the study sought answers from the HCWs as follows: (1) the hospital has been offering counselling services for its staff amid the COVID-19 pandemic (Counselling); (2) the hospital has been offering peer support groups sessions for its staff amid the COVID-19 pandemic (Staff support); (3) there are readily available coping strategies in the hospital for addressing my mental health concerns amid the COVID-19 pandemic (Coping strategies) and (4) the coping mechanisms offered by the hospital amid the COVID-19 pandemic have been beneficial in managing my mental health issues amid the COVID-19 pandemic (strategies beneficial; Figure 5). Each number in the grid shows the strength and direction of a relationship between two variables (Figure 5).

The spearman correlations ranges from −1 to +1, whereby +1 = perfect positive relationship meaning when one increases, so does the other; 0 = no relationship and −1 = perfect negative relationship meaning when one increases, the other decreases (Figure 5). Therefore with a correlation of 0.43 between staff support and coping strategy, which is a moderate positive relationship. The results imply that HCWs who feel more staff support tend to report better coping strategies. In addition a weak positive correlation of 0.29 was found between when comparing coping strategies and whether the strategies were beneficial. This imply that better coping strategies are somewhat linked to perceiving them as helpful by HCWs. Counselling with correlation scores between 0.00 and 0.12 did not have any meaningful relationship with other variables (Figure 5). Meaning counselling responses does not correlate with the other variables. Our results implies that the more staff support HCWs perceive, the better they cope and the more useful they find those coping strategies. However, counselling, does not show strong ties to those other experiences. This finding was surprising and in contrast with a lot of studies on mental health that recommend counselling and support of HCWs to assist them with mental health issues (Afzal et al., 2021; Al-Ashwal et al., 2020; Diehl et al., 2023; Rosen et al., 2020) In United States, Toronto hospital Rosen et al. (2020) reported that to bolster resilience amongst HCWs support and psychotherapy (which is counselling) are needed. In a published letter to the editor Afzal et al. (2021) recommended implementation of mandatory counseling sessions and availability of support centers for mental well-being of heath care workers during the COVID-19 pandemic. In Germany, Diehl et al. (2023) recommended counselling and support services for HCWs in German university hospitals during the pandemic. The HCWs of Yemen hospital showed a mean score of 25 (out of 30) for counselling practices amid the COVID-19 pandemic even though Yemen was war-torn then (Al-Ashwal et al., 2020). While our study results are surprising when compared to American and Asian case studies about, these findings are not unique in the African continent where governments are dealing with a lot of socio-economic issues, counselling and mental health issues are in most cases at the back of the mind of decision makers. In most cases in Africa, mental health issues are frowned upon in social circles and are therefore suppressed from most decision makers. In three African countries, local syndromes resembling nonpsychotic mental disorders were not regarded as a disorder (Ventevogel et al., 2013). Sankoh et al. (2018) reported that increased attention to mental health by governments, researchers, and journals is therefore essential in Africa.

## 4 Conclusion

### 4.1 Mental health measures

Figure 3 shows the intersectionality of mental health vulnerability, showing how language and caregiving roles interact with the emotional burden of a disaster. Therefore, targeted mental health interventions should focus on providing linguistically accessible services and outreach programs. In addition, support for caregivers, especially parents, through peer groups, counseling, and flexible work policies should be implemented by the Health Departments during pandemics such as the COVID-19 to support the HCWs to relieve some anxiety and compassionate fatigues level during pandemic times. From a disaster management perspective, these findings highlight the importance of incorporating social and cultural sensitivity into mental health and psychosocial support (MHPSS) frameworks during and after pandemics such as the COVID-19 pandemic.

The mental health measures findings from our study highlights the impact of language on anxiety levels and the burden of caregiving on compassionate fatigue. These insights should inform targeted interventions in public health messaging, community mental health programs, and disaster preparedness strategies. Proactive, inclusive, and equitable support mechanisms are necessary for effective crisis response and recovery especially in rural provinces across South Africa.

### 4.2 Institutional support

This study conclude that there is weak evidence of institutional support affecting HCWs’ training and preparedness to use mental health systems during COVID-19. Only promotion of a culture of self-care and mental well-being shows a borderline association, hinting that perceived promotion of self-care might influence whether HCWs feel adequately trained and equipped. The HCWs for this study did not consistently perceive institutional support as preparing them to manage mental health challenges during the COVID-19 pandemic. The only dimension that showed potential benefit was selfcare promotion. This highlights the importance of proactive, culturally embedded support strategies over reactive or logistical ones. For meaningful change, the Department of Health in South Africa must go beyond systems and embrace holistic, staff-centered approaches to mental well-being.

### 4.3 Coping strategies

In conclusion, the results highlight the critical role of staff support in influencing how individuals cope and how effective they perceive their coping methods. Investing in supportive staff environments may therefore enhance the overall effectiveness of mental health or well-being interventions. The lack of association with counselling might indicate a need to explore its delivery, accessibility, or perceived relevance among participants.

## Data Availability

The raw data supporting the conclusions of this article will be made available by the authors, without undue reservation.
