# Supplementary material for: Investigating the psychological impact of COVID-19 on Rob Ferreira Hospital healthcare workers in Mpumalanga Province, South Africa
Source: Front Psychol. 2025 Jul 29;16:1553866. doi: 10.3389/fpsyg.2025.1553866 (PMC12341473; doi:10.3389/fpsyg.2025.1553866)
Supplement: Supplementary file 1 [file Data_Sheet_1.docx]

**Healthcare Workers Questionnaire**

**Administration**

Dear Participant,

Your invaluable participation is sought for a research study focusing on the psychological impacts experienced by healthcare workers at Rob Ferreira Hospital in Mpumalanga Province, South Africa, amidst the COVID-19 pandemic. As frontline professionals, your insights and experiences are essential in providing a comprehensive understanding of the challenges and stressors faced in the course of your duties. This study aims to explore the depth and breadth of mental health challenges encountered by healthcare workers, including stress, anxiety, depression, and burnout, directly attributable to their roles during this global health crisis. By sharing your experiences and perspectives, you will contribute to the identification of key stressors, assessment of mental health conditions, and evaluation of existing support systems within the hospital. Your participation in surveys, interviews, and discussions is crucial in informing evidence-based interventions and support initiatives tailored to the unique needs of healthcare workers. Your input will enhance our understanding of the psychological effects of the pandemic and also contribute to the resilience and well-being among healthcare professionals during pandemics.

Thank you for considering participation in this important research endeavour. Your contribution will play a significant role in shaping the future of mental health support for healthcare workers at Rob Ferreira Hospital and beyond.

**Questionnaire No:**

**Section A: Demographic**

1. What is your age?
2. What is your gender?
3. What is your job title/position at Rob Ferreira Hospital?
4. How many years have you been working at Rob Ferreira Hospital?
5. In which department or unit do you primarily work?
6. What is your level of education?
7. What is your marital status?
8. What is your primary language spoken at home?
9. Do you have any dependents (e.g., children, elderly relatives)?
10. What is your current employment status (full-time, part-time, contractual, etc.)?

**Section B:** Mental Health Measures

1. I have experienced symptoms of stress (such as feeling overwhelmed or anxious) during the COVID-19 pandemic. What is your level of education?

| Yes |  |
| --- | --- |
| No |  |

1. I have experienced symptoms of depression (such as persistent sadness or loss of interest) during the COVID-19 pandemic. What is your marital status?

| Yes |  |
| --- | --- |
| No |  |

1. I have experienced symptoms of anxiety (such as restlessness or panic attacks) during the COVID-19 pandemic. What is your primary language spoken at home?

| Yes |  |
| --- | --- |
| No |  |

1. I have experienced symptoms of burnout (such as emotional exhaustion or detachment) during the COVID-19 pandemic. What is your current employment status (full-time, part-time, contractual, etc.)?

| Yes |  |
| --- | --- |
| No |  |

1. I have experienced symptoms of compassionate fatique (such as emotional and physical exhaustion resulting from prolonged exposure to others' suffering) during the COVID-19 pandemic. Do you have any dependents (e.g., children, elderly relatives)?

| Yes |  |
| --- | --- |
| No |  |

**Section C:** Institutional Support

1. I have been adequately trained and equipped to utilize the available support systems for addressing my mental health concerns amid the COVID-19 pandemic.

| Yes |  |
| --- | --- |
| No |  |

1. I have sought professional help or support for my mental health concerns during the COVID-19 pandemic

| Yes |  |
| --- | --- |
| No |  |

1. The existing support systems provided by the hospital management effectively address my psychological needs

| Yes |  |
| --- | --- |
| No |  |

1. The hospital's management encourages open communication and feedback regarding mental health issues among staff.

| Yes |  |
| --- | --- |
| No |  |

1. The hospital's management actively promotes a culture of self-care and mental well-being among healthcare workers.

| Yes |  |
| --- | --- |
| No |  |

**Section D: Coping Mechanisms**

1. Please indicate your level of agreement with the following statements by ticking the appropriate box:

| Statement | Strongly Agree | Agree | Not Sure | Disagree | Strongly Disagree |
| --- | --- | --- | --- | --- | --- |
| 21.1The hospital has been offering counselling services for its staff amid the COVID-19 pandemic | ☐ | ☐ | ☐ | ☐ | ☐ |
| 21.2The hospital has been offering staff support group sessions for its staff amid the COVID-19 pandemic. | ☐ | ☐ | ☐ | ☐ | ☐ |
| 21.3There are readily available coping mechanisms/strategies in the hospital for addressing my mental health concerns amid the COVID-19 pandemic. | ☐ | ☐ | ☐ | ☐ | ☐ |
| 21.4The coping mechanisms offered by the hospital amid the COVID-19 pandemic have been beneficial in managing my mental health issues. | ☐ | ☐ | ☐ | ☐ | ☐ |

**Thank You**
